# Regions of interest computed by SVM wrapped method for Alzheimer’s disease examination from segmented MRI

**DOI:** 10.3389/fnagi.2014.00020

**Published:** 2014-02-20

**Authors:** Antonio R. Hidalgo-Muñoz, Javier Ramírez, Juan M. Górriz, Pablo Padilla

**Affiliations:** Department of Signal Theory, Networking and Communications, University of GranadaGranada, Spain

**Keywords:** Alzheimer’s disease, gray and white matter, image segmentation, MRI, SVM

## Abstract

Accurate identification of the most relevant brain regions linked to Alzheimer’s disease (AD) is crucial in order to improve diagnosis techniques and to better understand this neurodegenerative process. For this purpose, statistical classification is suitable. In this work, a novel method based on support vector machine recursive feature elimination (SVM-RFE) is proposed to be applied on segmented brain MRI for detecting the most discriminant AD regions of interest (ROIs). The analyses are performed both on gray and white matter tissues, achieving up to 100% accuracy after classification and outperforming the results obtained by the standard *t*-test feature selection. The present method, applied on different subject sets, permits automatically determining high-resolution areas surrounding the hippocampal area without needing to divide the brain images according to any common template.

## INTRODUCTION

Alzheimer’s disease (AD) is a progressive, neurodegenerative disorder that gradually impairs memory and other cognitive skills, such as spatial orientation, judgment or language, preventing a healthy aging. Currently, scientists are interested in researching different kinds of brain imaging to detect possible dementia at a very early stage, when medical and psychological treatments are more effective. For Alzheimer’s disease, it is necessary to determine the brain regions of interest (ROIs); magnetic resonance imaging (MRI) provides valuable information on that matter. Several useful atlases and templates are reported in the literature (Shen et al., 2012). However, choosing the most appropriate one is difficult and it is not possible to use only one brain map to cover every specific characteristic from each neurological disorder. From a clinical standpoint, doctors generally require rigorous technical basis or mathematical methods (Rosenberg and Hillis, 2009). Therefore, a straightforward method is needed for discriminating the most relevant regions related to neurodegenerative diseases (López et al., 2011; Shen et al., 2011). In this study, an approach combining image segmentation and a wrapped classification algorithm is proposed for computing the ROIs from MRI, which differ meaningfully between AD patients and healthy elderly people.

Structural MRI has been widely explored in AD, giving valuable information about its underlying anatomical progression. For instance, the Alzheimer’s disease neuroimaging initiative (ADNI) has compiled an MRI database of AD, and mild cognitive impairment (MCI) that has been widely employed (Jack et al., 2010; Weiner et al., 2010). MRI and AD research goals vary. Several researchers in this field have focused on volume and integrity measurement of different brain tissues (Shen et al., 2011; Zhang et al., 2013) in order to find relevant early AD biomarkers or MCI onset (Desikan et al., 2009). Furthermore, much research attempts to achieve more efficient automated classification of AD patients as compared to MCI patients or healthy aging (Klöppel et al., 2008; Aguilar et al., 2013; Ortiz et al., 2013a). Overall, most of these studies determine the ROIs before the classification block according to previously delimited regions, which have been split based on any standard template (Mazziotta et al., 1995; Shattuck et al., 2008; Cuingnet et al., 2011).

Determining adequate brain ROIs is an important topic in medical image processing and computer-aided diagnostics (CAD) with many applications like morphology detection (Kapur et al., 1996) or 3-D visualizations for surgical planning (Suetens et al., 1993; Clarke et al., 1995). Usually, MRI is segmented into three quantitatively distinct tissues, that is, gray matter (GM), mainly linked to the cortex, white matter (WM), mainly composed by neuronal axons, and cerebrospinal fluid (CSF; Salas-Gonzalez et al., 2011). Despite the recent development of new MRI segmentation methods (Ortiz et al., 2013b; Salas-Gonzalez et al., 2013), the most recognized approach models intensity value distribution by a mixture of Gaussian distributions (MOG; Ashburner and Friston, 2003). This method is implemented in commonly used software like statistical parametric mapping (SPM; Frackowiak, 2004), which is utilized in diverse clinical protocols, and obtains the probability of each image voxel belonging to any tissue according to location and intensity level in a gray scale.

In general, machine-learning and classification techniques are increasingly used as an alternative to other multivariate statistical approaches. The aim of these pattern recognition techniques is not limited to achieving good results in classification tasks or for artificial intelligence applications, but rather to gage the relevance of some extracted features and search for differences between experimental conditions (Shieh and Yang, 2008; Hidalgo-Muñoz et al., 2013a,b; Tomé et al., 2013). Following this line, methods where the feature selection algorithm is wrapped around the classification algorithm recursively to identify the least relevant features constitutes a good option (Kohavi and John, 1997). These algorithms are suitable for dealing with high-dimensional data like medical images, since the parameters of the classifier serve as scores to select the ROIs and the corresponding classification performance guides the iterative procedure. On the other hand, generalizing results will depend on the size of the dataset, and the cross-validation (CV) method used to evaluate the classification accuracy (Burges, 1998). Either way, these methods permit covering a whole set of initial features without being restricted to any specific region to check its relevance. In this work, the recursive feature elimination (RFE) algorithm, proposed by Guyon et al. (2002), and based on the support vector machine (SVM; Ben-Hur et al., 2008), is used. Support vector machine recursive feature elimination (SVM-RFE) has been successfully implemented in various neuroscience applications (De Martino et al., 2008; Chu et al., 2012; Hidalgo-Muñoz et al., 2013a); nevertheless, it has hardly been used for image analyses.

The presented work focuses separately on GM and WM tissues to delimit the most discriminant brain ROIs for examining AD from MRI. This paper presents an innovative and effective method for feature selection, the SVM-RFE technique, that has never been used before for this purpose as far as authors are aware. This affordable and intuitive method, easily implementable in medical apparatuses, intends to contribute to a complete diagnosis and examination of AD and its progression.

## MATERIALS AND METHODS

### DATASET

Data used in the preparation of this article was obtained from the (ADNI) database (http://adni.loni.usc.edu/). The ADNI was launched in 2003 by the National Institute on Aging (NIA), the National Institute of Biomedical Imaging and Bioengineering (NIBIB), the Food and Drug Administration (FDA), private pharmaceutical companies and non-profit organizations, as a $60 million, 5-year public-private partnership. The primary goal of ADNI has been to test whether serial MRI, positron emission tomography (PET), other biological markers, and the progression of MCI, and early AD. Determining sensitive and specific markers of very early AD progression is intended to aid researchers and clinicians to develop new treatments, as well as reduce the time and cost of clinical trials. The Principal Investigator of this initiative is Michael W. Weiner, MD, VA Medical Center and University of California, San Francisco. ADNI is the result of efforts of many co-investigators from a broad range of academic institutions and private corporations, and subjects have been recruited from over 50 sites across the U.S. and Canada. The initial goal of ADNI was to recruit 800 adults, ages 55 to 90, to participate in the research: approximately 200 cognitively normal older individuals to be followed for three years, 400 people with MCI to be followed for three years and 200 people with early AD to be followed for two years. For up-to-date information, see *www.adni-info.org*.

In this article, only the data from T1-weighted MR images was considered. The participants were separated into two different classes:

– *Normal.* Control subjects. Clinical Dementia Rating (CDR; Morris, 1993) of zero. They were non-depressed, non-MCI and non-demented.

– *AD.* CDR of 0.5 or 1, met NINCDS/ADRDA criteria for probable AD (McKhann et al., 2011).

**Table 1** shows the demographic details of the subjects who compose the dataset used in this work.

**Table 1:** Sociodemographic data.

Group	Subjects	Sex: M/F	Age:μ(SD)	MMSE:μ(SD)
Normal	185	95/90	75.85(5.11)	29.15(0.97)
AD	185	98/87	75.39(7.56)	23.28(2.05)
*p*	-	-	*0.489*	*<0.001*

*
MMSE: mini-mental state examination (Folstein et al., 1975). μ: mean; SD: standard deviation; *p*: significance of *t*-test contrast between-groups.*

### IMAGE PRE-PROCESSING

The SPM software was originally designed for analyzing functional brain images. The package also contains routines for realignment, smoothing, and spatial normalization into a standard space of T1-weighted MR images. To this end, the template implemented within the VBM8 Toolbox was used, specifically DARTEL, to achieve an accurate realignment of the images and a good normalization (dbm.neuro.uni-jena.de/vbm/). It is worthwhile to stress that spatial normalization or the registration algorithm is always a critical component to any classifier that uses voxel-wise features (Cuadra et al., 2005). Within these routines, a modulation step was implemented in order to conserve the amount of tissue and not the intensities (see Ashburner et al., 2012; p. 192). After the transformation of the images from the ADNI database, they were resized to the dimensions 121 × 145 × 121 with voxel sizes of 1.5 mm (sagittal) × 1.5 mm (coronal) × 1.5 mm (axial). Neither smoothing nor dimension reduction were performed after that. One high-quality image per subject was chosen.

### IMAGE SEGMENTATION

In this work, GM and WM image segmentation was implemented in SPM, which models the intensity value distribution of the T1-weighted MRI by a MOG (Ashburner and Friston, 2003, 2005) and takes voxel location into consideration via a tissue probability map (TPM). Using this methodology, which is described, for example, in Ashburner et al. (2012), it has been possible to overcome the partial volume effect (PVE), such that a voxel may not be purely of one tissue class, but can contain signals from a number of different tissues.

Within this work the central features are the probability values for GM or WM in a given voxel, not the intensity values *per se*. Once the images have been segmented, the resulting dataset is ready for further processing and analysis. The subsequent analyses were carried out with MATLAB^TM^.

### SVM WRAPPED METHOD

As mentioned, medical images may provide clinicians valuable information about disease status, diagnosis and prognosis. However, extracting significant features from high-dimensional data as an image is always a complex task. Usually, some reduction methods, and their subsequent feature selection, greatly transform the original data, making eventual clinical interpretations difficult. The aim of this paper is to select a reasonable number of ROIs with high predictive value and whose aspect could then be medically meaningful.

In the wrapper approach, the feature selection algorithm wraps around the classification algorithm. The feature selection consists of searching high-dimensional data sets using the induction algorithm itself as part of the evaluating function (Kohavi and John, 1997). Hence the parameters of the classifier serve as scores to select (or to eliminate) features; the consequent classification performance guides an iterative procedure. When this recursive feature elimination strategy uses a linear SVM-based classifier, the resulting method is known as support vector machine-recursive feature elimination (SVM-RFE; Guyon et al., 2002). The validation method will be crucial to avoiding creating a system that is over-trained, that is, that fits well only to the experimental data, losing generality and providing misleading results.

An SVM-based classifier separates a given set of binary-labeled training data with what is known as the maximal margin hyperplane, which is maximally distant from two classes (for example, *AD* and *Normal* classes). The objective is to build a function that will correctly classify new examples (for example, *MRI-segmented images*).

Linear SVM parameters define a decision hyperplane in the multidimensional feature space (Burges, 1998; Ben-Hur et al., 2008; Illán et al., 2011), that is:

$$g\left(x\right)=w^{T}x+b=0$$

where **x **denotes the feature vector, **w **is known as the weight vector and *b *is known as the threshold.**The decision hyperplane position is determined by vector **w **and *b*: the vector is orthogonal to the decision plane and *b *determines its distance to the origin. For linear SVM, the vector **w **can be explicitly computed. The design of the classifier consists of finding the unknown parameters, that is, *w* components of **w** (*w*_n_, n = 1…M, where* M = *number of features) and* b, *which allows building a hyperplane that separates the two classes optimally.

**FIGURE 1:**
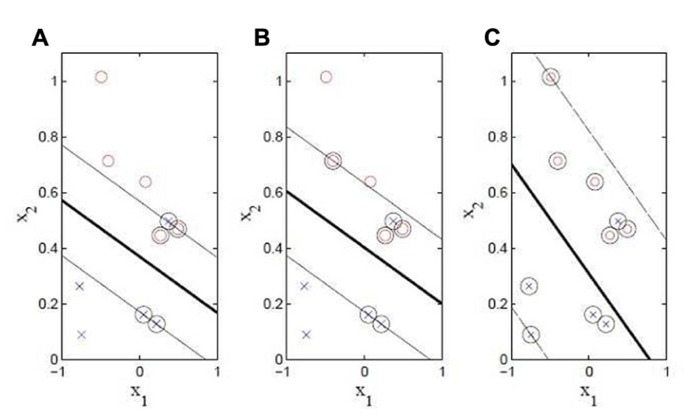
**SVM schemes using different *C* values.** **(A)** C =100. **(B)** C =1. **(C)** C =0.1. Thicker line: decision lines (hyperplane). Thinner lines: margin limits depending on the *C* parameter.

**Figure 1** illustrates a 2D toy-example of a binary classification problem, where the points x=[x_1_ x_2_], marked like red circles, belong to one class, and the ones marked like blue crosses belong to the other one. The problem is not linearly separable since it is not possible to find a line (2D hyperplane) that perfectly separates all training instances of the two classes. However, if a small number of misclassifications are tolerated, the problem becomes linearly separable. The figure shows the result of three training sessions with the same data, but different misclassification margins (*C* parameter). The vector $w=\sum_{1}^{N_{s}}y_{i}\lambda_{i}X_{i}$ is a weighted sum of the *support vectors* which are the *N*_s_ elements, inside the margin, chosen from the set used during the training phase. In **Figure 1**, these support vectors are marked with circles around the training data points. λ_i_ are the corresponding Lagrangian parameters which are also optimized (0 <λ_i_ <*C*). Finally, the value of the threshold *b* is estimated by solving the equations related to the hyperplanes that define the margin. In Ben-Hur et al. (2008), an extensive algebraic explanation of SVM applied to biological sciences is reported.

The value of* C* must be assigned to run the optimization algorithm and represents the weight of the penalty term of the function that is related with the training set misclassification error. It is a parameter that indirectly controls the margin width of the classifier (see **Figure 1**). A trade-off exists between the width of the margin and the number of accepted misclassifications. There is no optimal procedure to assign this parameter, but it has to be expected that if *C* is large, the misclassification errors are relevant during the optimization function and the margin should be narrow. On the other hand, if *C* is small, the misclassification errors are not relevant and a large margin has is expected.

**FIGURE 2:**
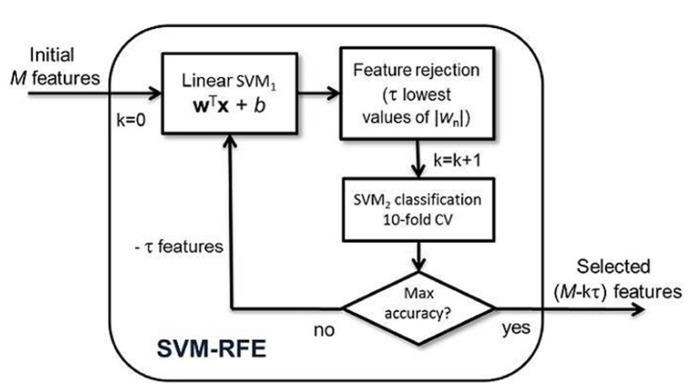
**SVM-RFE scheme.** SVM_1_ parameters are computed taking all the available samples for the training block and determining the relevance of the features. SVM_2_ classification is performed in order to check the suitability of the previous selected features by SVM_1_ and serves as a guide to achieve the optimal number of features. CV: Cross-validation.

According to SVM-RFE algorithm, the relevance of the feature vector’s *n*–*th *entry is determined by the corresponding value *w*_n_ in the weight vector. In particular, if |*w*_n_| ≃ 0, the corresponding feature does not contribute significantly for the value of *g*(x). Then, sorting these absolute values, the relevance of the features is determined. Therefore, in each loop of the algorithm, a concrete number of features (τ) can be discarded following this criterion (see **Figure 2**).

The method explained above is closely linked to the principle of the nested CV approach for classifier optimization (Varma and Simon, 2006; Gallix et al., 2012). The nested CV techniques use loops into the training blocks for designing and tuning the classifier’s parameters (see **Figure 2**). Therefore, following the recommendations reported in the literature (Varma and Simon, 2006), validating the complete algorithm was carried out on different subsets from the initial whole sample in order to diminish biased results (see section 2.5).

As described in the previous section, the feature values after segmentation represent the probability of belonging to the tissue (GM or WM) of every voxel of the image. Therefore, the initial set of features, using a voxel-as-feature (VAF) approach, is composed of 121 × 145 × 121 = 2,122,945 voxels from each original image. Then, the number of features is reduced for each classification task, since only those from the separated segments were used as algorithm inputs. As a result, the number of initial features for the GM class (*M *= 568,273 voxels) was slightly higher than for the WM class (*M *= 504,329 voxels).

Assuming the trade-off between resolution and computational cost, after empirical trials, a step (τ) of 20,000 features to be eliminated in each SVM-RFE loop (approximately 3.5–4% of the total number of features) was considered as a proper option in the experiments.

### PERFORMANCE MEASUREMENT

As indicated in **Table 1**, a total of 370 subjects (185 *AD* and 185 *Normal*) were employed in the experiment. However, in order to reduce the computational cost and get a more reliable generalization, several subsets were formed from the original whole set to carry out selection and classification (both GM and WM alternatives). The sets were composed of 60 subjects (30 *AD* and 30 *Normal*).

The subjects were organized randomly and the first 30 subjects belonging to each group (*AD* or *Normal*) were taken as the first set. Then, a “sliding window” with an overlap of 25 subjects for each group went across all the subjects, forming new groups. Thus, a total of 32 different sets were constituted to carry out the mentioned selection and classification tasks. Finally, the global accuracy of the method was computed by averaging the partial accuracies obtained using each set. Additionally, by applying SVM-RFE on different sets, the feature selection permits quantifying more exhaustively the extent to which the results are generalizable. Obviously, there were subjects that belonged to several groups and some redundance was unavoidable. Once the main features were selected by the first blocks of SVM-RFE, an independent SVM classification task was performed using 10-fold as a CV strategy for evaluating the classifier accuracy (see SVM_2_ in **Figure 2**), which is a suitable method for diminishing image variability or peculiarity influence and has been strongly suggested in machine-learning applications (Kohavi, 1995; Ortiz et al., 2013a). The 10–fold CV consists of using all the samples in each subset for training the system except 10, which is used as a test. This procedure is repeated S times, S being the number of 10–sample groups in the dataset, after which a global accuracy value is computed.

### STATISTICAL ANALYSIS

In order to check the statistical significance of the differences between voxels belonging to the averaged images from the *AD* and *Normal* groups, Student’s *t*-tests for independent samples were performed, assuming a significance level of *p *= 0.05, which is widely used in brain image processing (Du et al., 2001; Liu et al., 2010; Hölzel et al., 2011; Li et al., 2011).

Moreover, a study on the different sets’ global accuracy was carried out, computing the mean and standard deviation (SD) for each loop of the algorithm.

## RESULTS

### REGIONAL DIFFERENCES IN GM

**FIGURE 3:**
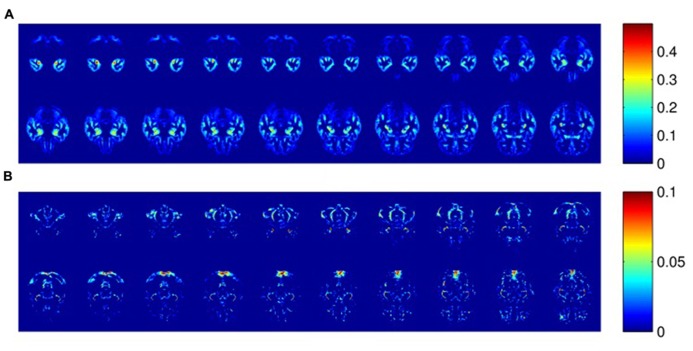
**Averaged differences in the probabilities of belonging to GM between *AD* and *Normal* groups.** Horizontal sections (z axis, 23 <z <42). Color bars represent these differences. **(A)** ROIs where the GM probability is higher in *Normal* than *AD* (maximal difference =0.5). **(B)** ROIs where the GM probability is higher in *AD* than *Normal* (maximal difference =0.1).

**Figure 3** shows the differences in representative axial sections from GM segmented images between *AD* and *Normal* groups in order to get a rough idea about the location of the most evident ROIs. Only GM segmentation is represented, which is emphasized in this kind of research, since it is only for visualization purposes.

### ROIs ACCORDING TO *t*-TEST (GM AND WM)

Using the significant features determined by the *t*-test threshold, the obtained accuracy applying the same procedure yielded 78.66% for GM and 77.8% for WM. Applying the *t*-test on the complete sample (a total of 185 subjects for each group to compute the contrast and performing only one global classification task after the feature selection, 10–fold validation), the accuracy improved to 89.46% for GM and 93.24% for WM, although these values are still lower than those obtained by SVM-RFE, as described in the next section. Furthermore, if only the top 20% of the relevant features according to the *t*-test (and corresponding *p* values) was employed, the accuracy decreased to 63.98% and 74.59% for GM, applying the same CV method explained in section 2.5 (32 sets) and using the complete sample, respectively, and 77.85% and 93.24% for WM. Note that in the case of WM, the results are similar taken specifically the significant features or only the 20% because of the number of significant features (*p *<0.05) matched up approximately to the 20% of the total features.

## ROIs ACCORDING TO SVM-RFE (GM AND WM)

**FIGURE 4:**
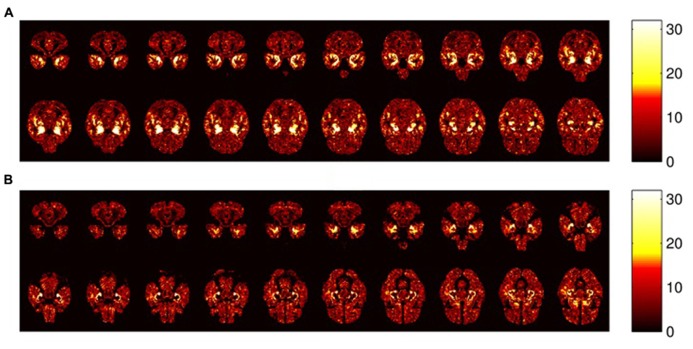
**Representative axial sections (z axis, 23 <z <42) where relevant differences have been found that delimit ROIs by SVM-RFE.** Only the 20% most-relevant features are taken into account. Color bars represent the number of training sets that select each feature. **(A)** GM segmentation. **(B)** WM segmentation. Note that the most brilliant regions are the most relevant.

**Figure 4** shows the most relevant ROIs (GM and WM segmented images) according to the remaining features after applying SVM-RFE using multiple groups for the algorithm training (see section 2.5). According to the accuracy values obtained in both classification tasks, and intending to get a representative amount of features to make illustrative comparisons, only the top 20% most-relevant features pointed by SVM-RFE were chosen as ROIs.

**FIGURE 5:**
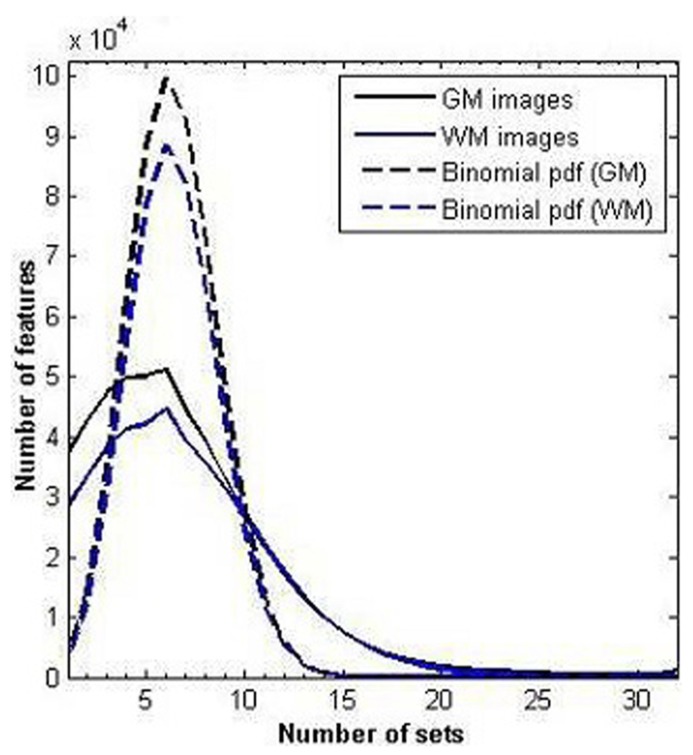
**Number of times that specific features have been selected as relevant ones from the total of the 32 sets.** Solid lines represent the result obtained by applying SVM-RFE; dash lines represent the corresponding binomial PDF for both conditions.

The number of features considered relevant by diverse number of sets is represented in **Figure 5**. In **Figure 5**, two binomial probability density functions (PDFs) are represented as well, whose parameters are *n *= 32 (number of sets) and *p *= 0.2 (success probability being considered as relevant). If the feature selection by every set had been random and independent of the rest of the sets’ decisions and the features had the same probability of being chosen and were statistically independent as well, these binomial PDF shapes would have been expected in the graphs.

Note that SVM-RFE leads to a substantial reduction in the number of relevant features. Either way, if the target of this work had been to design a potential classifier, instead of computing ROIs, other external data should have been tested.

**FIGURE 6:**
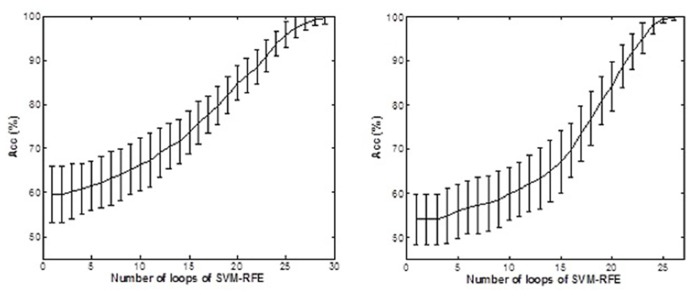
**Accuracy values (%) for (A) GM and **(B)** WM segmentations by applying SVM-RFE**.

**Figure 6** shows the accuracy values and standard deviations of the *AD* vs. *Normal* classification tasks, selecting the relevant features basing on SVM-RFE. The maximum averaged accuracy values were 99.64% using GM images and 99.74% using WM images.

Since the objective of the method is to achieve maximum accuracy (practically 100%) in order to discriminate the ROIs, it is not necessary to include either sensitive or specificity measures, in contrast to other applications whose goals focus on designing the best classifiers and clinical repercussions of their errors.

In order to check again the method’s generalization capability, a global classification task was performed after the previous feature selection by SVM-RFE. In this classification task, different amounts of features were chosen based on different required percentages of sets that indicated features as belonging to the 20% most-relevant ones. In other words, the features were grouped depending on how many times they were selected by all the sets. In this case, the complete sample was considered (185 subjects per group) and 10-fold validation method applied. The results suggested an acceptable generalization, since 94.32% accuracy was reached for GM images taking into consideration only those features selected as relevant by at least 18 of the 32 sets (56%of the sets). However, the accuracy value decreases to 82.16% if only those features selected by 100% of the sets are chosen because too few features are reckoned in that case. Similar results occur with WM segmented images, where 95.14% accuracy is achieved when the features selected by at least 14 sets (44% of the sets) are chosen. However, it is important to note that, in this case, the 10-fold validation method is not a strict CV, since the previous ROIs were selected using the complete sample. Either way, the results can give an idea about potential reproducibility.

### 3-D ILLUSTRATIONS

**FIGURE 7:**
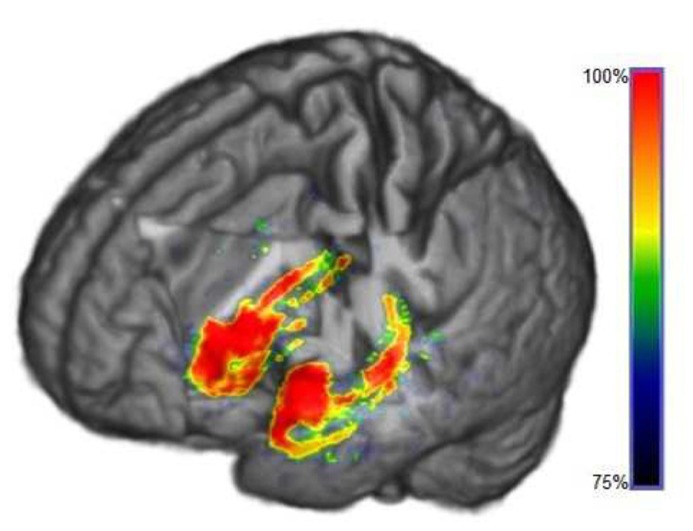
**3-D illustration of the ROIs determined by SVM-RFE algorithm in GM segmented MRI selected at least by 75% of training sets to 100%**.

**FIGURE 8:**
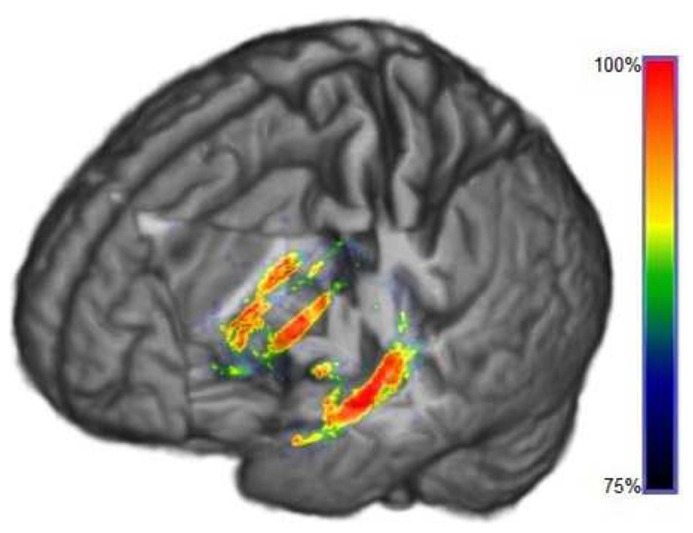
**3-D illustration of the ROIs determined by SVM-RFE algorithm in WM segmented MRI selected at least by 75% of training sets to 100%**.

**Figures 7 and 8** show three-dimensional illustrations of the ROIs computed by the SVM-RFE method for GM and WM, respectively. In the graphs are marked only those voxels that were selected by the majority of the training sets (more than 75% of the sets’ total) in order to show the most robust results.

## DISCUSSION

In this work, a novel application of a wrapper method based on SVM was performed to determine the ROIs from MRI, belonging to both GM and WM tissues, in order to better detect AD. Neither reduction of the data dimensionality nor previous voxel selection was required, thereby keeping the highest possible resolution to delimit the mentioned ROIs (see **Figures 7 and 8**).

From the beginning, the SVM-RFE algorithm was widely used in studies in order to select important genes (Guyon et al., 2002; Tang et al., 2007; Mundra and Rajapakse, 2010). Nonetheless, its potential applications have hardly been considered for image processing and specifically for ROIs computing from brain images. In this paper, SVM-RFE has been shown to be able to select relevant ROIs that may aid an early diagnosis of AD. This is achieved by means of using the probabilities of belonging to GM or WM tissues of each voxel as features, which composes a segmented MRI. Methods based on SVM classifiers are suitable for analyzing big data, such as image processing, where the curse of dimensionality is a very common concern, since the SVM algorithm is not sensitive to over-fitting thanks to its margin definition (*C* parameter). In any case, it is fair to reiterate this concern to be cautious for the generalization of this kind of results.

The results obtained in the presented experiments have suggested that SVM-RFE selects discriminant features more efficiently than *t*-test significance for classification purposes (see section 3.4), as other research works report (Hidalgo-Muñoz et al., 2013a), specifically in digital signal processing. Furthermore, since wrapped algorithms are based on the same classification technique, the complete method becomes more simple and intuitive than other multivariate statistical approaches. Moreover, the SVM-RFE permits choosing features from a huge set, without needing the multiple comparison corrections that are still a controversial issue in statistics (Perneger, 1998). Either way, the CV method into the wrapper algorithm is a key block for evaluating the accuracy and testing the capability of classifier generalization.

Regarding region morphology, ROIs delimited by SVM-RFE are located mainly in specific regions where more differences are evident by comparing the MRI averages, matching up considerably within the expected regions that are statistically different between groups (see **Figures 3 and 4**). However, the ROIs are not limited to the statistically significant regions, but also cover voxels surrounding these regions and disregard numerous voxels inside them. These regions’ borders could be particularly important, since they could be related to the cortical atrophy suffered by AD patients in early stages (Quiroz et al., 2013). However, it would be necessary to check individually whether the ROI differences are mainly due to tissue deterioration (especially GM) or if they are influenced by some internal heterogeneity of the matter. For this reason, maintaining high MRI resolution is essential. This fact could question the suitability of some predefined ROIs by general brain templates (Magnin et al., 2009; Shen et al., 2011) in machine-learning applications that deal with such concrete neurological disorders as AD, whereas SVM-RFE takes all the features from MRI as a whole set without preconceived areas of interest.

The ROIs computed by SVM-RFE correspond intimately to the regions pointed in the literature as most affected by AD: hippocampus, entorrhinal cortex, parahippocampal region (Du et al., 2001; Velayudhan et al., 2013) and insular cortex (Xie et al., 2012) among other regions like the amygdala, lenticular nucleus or fusiform gyrus (Tzourio-Mazoyer et al., 2002). As is well known, the hippocampus is a basic subcortical structure involved in declarative memory consolidation and spatial orientation (Squire, 1992; Tsien et al., 1996), which are skills severely affected by AD. These regions show an evident inter-hemisphere symmetry, particularly for GM segments (see **Figures 4 and 7**).

In addition, **Figures 4 and 7** show that although neocortex deterioration is often evident in AD (Quiroz et al., 2013), it is possible to point out a more general pattern for all patients in temporal lobes and insular cortex, whereas frontal lobe damage seems to be more diffuse in this inter-subject study. This fact can be influenced by the images’ high-resolution, the necessary pre-processing and the specific and different development of the frontal lobes in people, depending on their experiences and even their educational level (Stuss and Knight, 2013) suggesting a remarkable contribution of individual differences.

The 3-D illustration helps to understand the possible generalization of the results for aid diagnosis or for clinical evaluations. Once the first ROIs have been determined, it would be possible to focus on these regions exclusively and separately in order to enclose and analyze in detail the pertinent anatomical regions (Ahmed et al., 2013). For instance, some research suggests that distinct regions of the hippocampus are affected differently in AD (Burger, 2010; Lee et al., 2012). Therefore, a rigorous study using SVM-RFE might be recommended for exhaustive anatomical inspections and analyzing particular cases. On the other hand, by maintaining the high-resolution, it would be possible to examine some regions that are difficult to delimit in averaged images due to their location and size, such as the *locus coeruleus *or the fornix.

Regarding the comparison between GM and WM, the former tissue provides more relevant information and delimits more properly the important ROIs, as is suggested in much research that analyzes the GM directly (Thompson et al., 2003; Karas et al., 2004). Whereas for GM segmentation there are more highlighted regions using SVM-RFE, for WM segmentation the ROIs are distributed vaguely similarly to the results obtained with *t*-test. In any case, by means of the proposed methodology, it is possible to achieve practically 100% accuracy on average for both GM and WM segmentation options (**Figure 6**) after running a complete SVM-RFE in different feature sets.

In future works, it could be suggested to apply the same method to GM and WM segments obtained by other sophisticated MRI processing approaches. In addition, it is possible to employ the method in artificial intelligence applications in medicine for investigating diverse neurological disorders linked to senescence, such as fronto-temporal lobar degeneration, different forms of dementia or Parkinson’s disease among others, where an accurate MRI information management is crucial.

## CONCLUSION

In this paper, the main region of brain interest involved in Alzheimer’s disease has been delimited by means of a SVM-based wrapper method applied on structural images, that is, MRI. The proposed method, which recursively eliminates the least-relevant features from the initial set (SVM-RFE), has proven to outperform *t*-test selection in terms of accuracy, achieving practically 100%. The high-resolution ROIs have been computed for both gray and white segmented matters, matching up with recent research that designates the hippocampal region as one of the most important in Alzheimer’s disease development. In addition, 3-D illustrations of the regions have been provided in order to better understand the anatomical morphology linked to AD. Furthermore, this method, previously unexplored for MRI, could give valuable information about brain structures in other clinical applications on aging research.

## AUTHOR CONTRIBUTIONS

Data used in preparation of this article was obtained from the Alzheimer’s Disease Neuroimaging Initiative (ADNI) database (http://adni.loni.usc.edu/). As such, the investigators within the ADNI contributed to the design and implementation of ADNI and/or provided data, but did not participate in the analysis or writing of this report. 

## Conflict of Interest Statement

The authors declare that the research was conducted in the absence of any commercial or financial relationships that could be construed as a potential conflict of interest.
